# Pediatric laparoscopic partial cystectomy for the treatment of benign bladder tumors and urachal cysts

**DOI:** 10.1186/s12894-021-00893-6

**Published:** 2021-09-15

**Authors:** Hongjie Gao, Jiawei Chen, Guowei Li, Xinhai Cui, Fengyin Sun

**Affiliations:** 1 Department of Pediatrics, Qilu Hospital of Shandong University, Jinan, China; 2 Department of Pediatric Surgery, Qilu Hospital of Shandong University, Jinan, China

**Keywords:** Laparoscopy, Partial cystectomy, Benign bladder tumor, Urachal cyst

## Abstract

**Objective:**

To investigate the feasibility and efficacy of carrying out pediatric laparoscopic partial cystectomies (LPC) when treating benign bladder tumors and urachal cysts.

**Methods:**

Retrospectivey analyzing 4 clinical cases involving children with bladder tumors, which were collected from October 2017 to December 2018. In these clinical cases, there were 3 male children and 1 female child, aged from 4.5 to 9.4 years old, with an average age of 6.5 years. An intraperitoneal laparoscopic partial cystectomy was performed in the treatment of 3 of these patients with benign bladder tumors and in 1 patient with an urachal cyst. The surgical procedures included a partial cystectomy and a complete intracavitary bladder suture.

**Results:**

All 4 cases were successful and no operation was transferred to opensurgery. The operation time was 100–120 min, with an average time of 108 min. The intraoperative blood loss was 10–20 ml, with an average loss of 15 ml. 6 h after the operation, the patients still maintained a fluid diet and 1 case of hematuria had occurred, with the catheter removed 12 days after the operation. No postoperative urine leakage, intestinal adhesion or intestinal obstruction occurred, and the average postoperative hospitalization time was 14 days.

**Conclusion:**

Laparoscopic partial cystectomy is a safe and feasible method to be used for the treatment of benign bladder tumors and urachal cysts. It presents the advantages of being minimally invasive, and having a quick recovery and short hospitalization time. It is an alternative surgical method for the treatment of pediatric benign bladder tumors.

## Background

Pediatric bladder tumors are infrequent, with rhabdomyosarcoma (RMS) being the most common. Benign tumors are even less common. They have various pathological types, and usually come to light in case reports. Therefore, clinicians do not know enough about them. In order to improve the diagnosis and treatment of pediatric benign bladder tumors then, we have retrospectively analyzed the clinical data from 4 pediatric benign bladder tumors that were admitted to our hospital from October 2017 to December 2018. From here, we have summarized the surgical experience and the indications.

## Materials and methods

### Clinical data

**Fig. 1:**
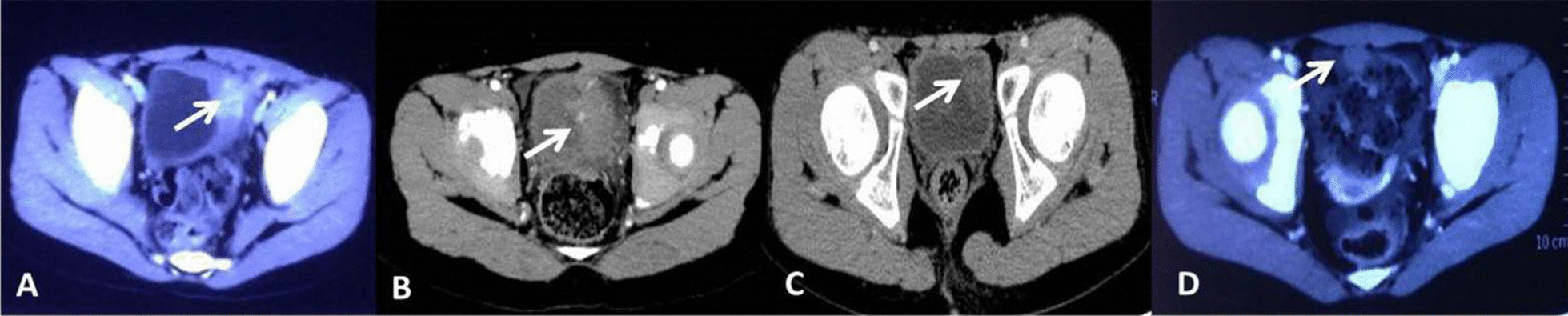
Photographs of CT scan: Patient 1 (**a**), Patient 2 (**b**), Patient 3 (**c**), Patient 4 (**d**); the arrow points to the tumor

**Fig. 2:**
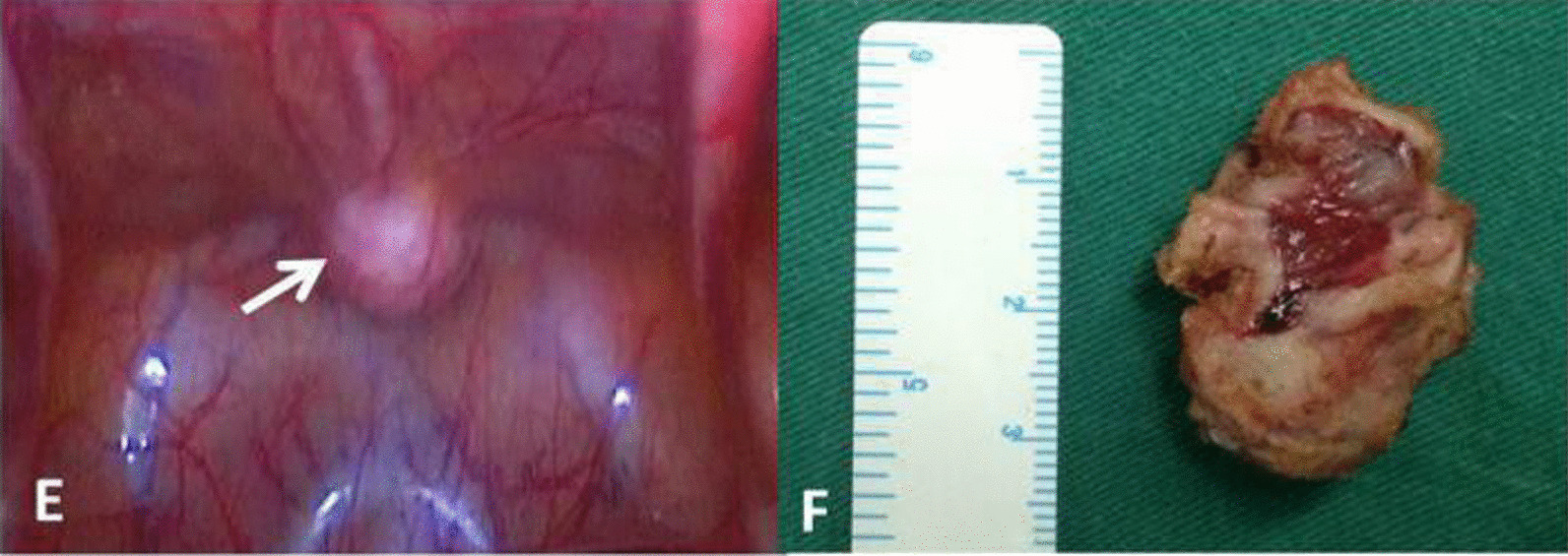
Photographs of laparoscopic Urachal cyst (**e**), postoperative Urachal cyst (**f**)

There were 4 cases in this group, including 3 males and 1 female, aged from 4.5 to 9.4 years old, with an average age of 6.5 years. Before the operation, a urinary b-ultrasound, pelvic CT and cystoscopy, as well as other examinations were all performed. The lesions were located in the apical wall in 1 case, the bottom in another case and the lateral wall in the remaining 2 cases, while all patients had symptoms of endless urination and bladder irritation, but no hematuria. In 2 cases, the left lateral wall had significantly thickened and there were rounded nodules on its top part. The CT showed sharp angular tissue shadows above the bladder in 1 case, while the urachus was not closed. Cystoscopy: in 3 cases, semicircular or irregular bulges were observed locally on the bladder wall, but the surface mucosa was smooth and continuous (Table 1, Figs. 1, 2).

**Table 1:** Perioperative patient data

	Age (yrs)	Gender	Clinical symptom	CT	Cytoscopic examination
Patient 1	4.7	Male	Odynuria 20 days	The left lateral wall is markedly thickened, at top of the left lateral wall are rounded enhanced nodules	There is a 5.2 cm irregular lump on the top of posterior wall, the basilar part is broad, from the ureterostoma 1–2 cm
Patient 2	4.6	Male	Micturition unwell 9 days	The left bladder wall is diffuse thickening, an irregular soft tissue mass protruded into the bladder	There is a 4.32 cm cauliflower-like lump on the left wall, from the ureterostoma about 3 cm
Patient 3	9.5	Male	Ultrasound found space occupying 1 month	The left side wall is thickened with clear boundary and irregular shape	There is a 2 cm cauliflower-like lump on the left of the bottom wall, the basilar part is broad, from the ureterostoma about 3.5 cm
Patient 4	7.3	Female	Paroxysm celialgia half month	A sharp angular shadow of tissue is seen above the bladder, considering urachal duct isn’t close

## Methods

The study was approved by the research ethics committee of Qilu Hospital of Shandong University, shandong, china. All methods were carried out in accordance with the relevant guidelines and regulations. A parent of each participant provided their informed consent for their child to participate in the study. Informed consent for the publication of this study was also obtained from the parents of all participants in the study.

**Fig. 3:**
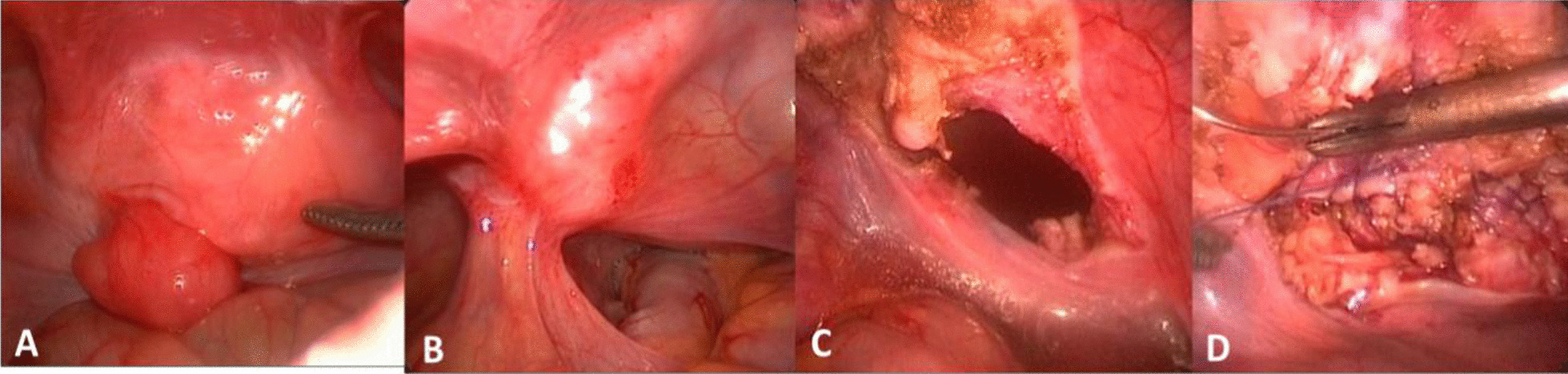
Photographs of laparoscopic bladder exogenic tumor (**a**), bladder endogenous tumor (**b**), and bladder wound (**c**) and suture of bladder wound (**d**)

After administering a satisfactory quantity of anesthesia, place a small pillow under the buttocks and keep the patient in the Trendelenburg position. Routinely disinfect the abdomen and perineum, and after operating, place the inserted urinary tube on the table. Following this, cut open the umbilical region, directly place a 5 mm trocar as the observation hole, fill with CO_2_, and maintain the pneumoperitoneum pressure at 8–10 mmHg. Place 5 mm trocars in the left lower abdomen and right lower abdomen under laparoscopy. Through the catheter, the bladder should be filled with an appropriate amount of germfree saline. Following this, through a combination of preoperative imaging and cystoscopy results, the tumor boundaries can be clearly distinguished under laparoscopy. Next, cut open the bladder wall at a position about 0.5 cm from the tumor, and then completely remove the tumor at a position of 0.5 cm around the tumor while under direct vision. Immediately put the specimen into the specimen bag, put it in the iliac fossa, full-thickness suture the incision with 4–0 absorbable suture, and embed it with interrupted seromuscular suture. Place the drainage tube outside the bladder wall at the site of the tumor resection, remove the instruments, suture the incision, and indwell the catheter (Fig. 3).

**Fig. 4:**
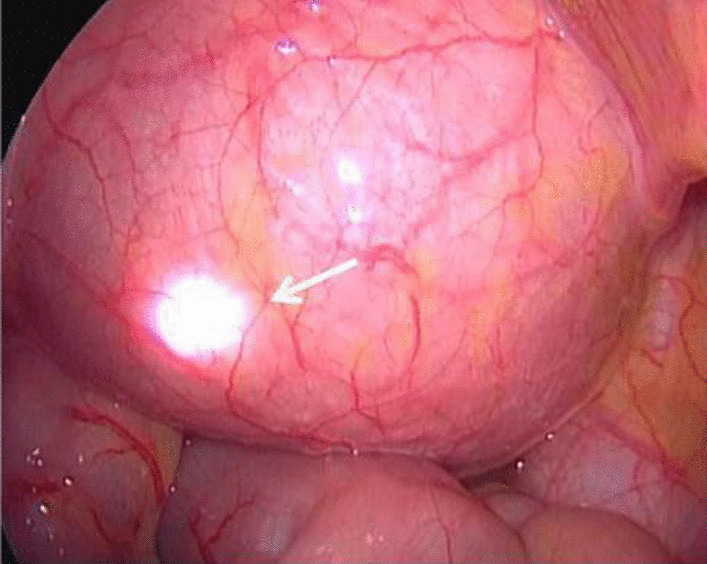
Double lenses combination method, arrow indicates cystoscope light source

Tumor location: (1) an exogenous tumor’s position can be identified under direct vision. (2) An infiltrative tumor can be located according to the degree of muscle layer thickening, or can be observed by opening the bladder and putting a lens in, whereby the location, size, and the relationship with the bilateral ureteral openings can be directly observed. (3) Using the double lens combination method, whereby a cystoscope is placed through the urethra at a distance of about 0.5 cm from the edge of the tumor. The brightness of the laparoscopic light source is reduced, and the location of the light source of the cystoscope is confirmed, where upon it should be marked with an electric hook (Fig. 4).

## Results

**Fig. 5:**
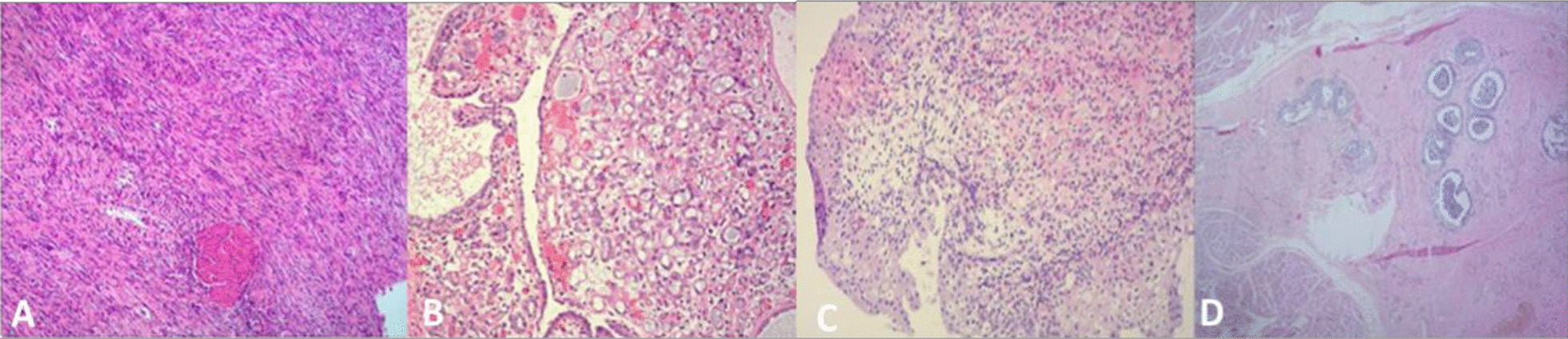
Photomicrograph of resected bladder lesion showing inflammatory myofibroblastoma (**a**), adenomatoid metaplasia (**b**), inflammatory granuloma (**c**) and urachanl cyst (**d**)

All 4 cases were successful and no operation was transferred to open surgery. The operation time was 100–120 min, with an average time of 108 min. The intraoperative blood loss was 10–20 ml, with an average loss of 15 ml. The patients maintained a fluid diet for 6 h after the operation. Hematuria occurred in 1 case. The catheter was removed 12 days after the operation. No postoperative urine leakage, intestinal adhesion or intestinal obstruction occurred, and the average time of postoperative hospitalization was 14 days. Postoperative pathological specimens results: 1 case was inflammatory myofibroblastoma, 1 case was adenomatoid or middle renal metaplasia, 1 case was reepithelialized granulation tissue, and 1 case was an urachal cyst. The 4 patients were followed up with for a period of 3–15 months, with an average follow-up of 10.4 months. There was no infection, recurrence or canceration, and the incision healed well by normosthenuria. (Table 2, Fig. 5).

**Table 2:** Postoperative and pathological data

	Operative time (min)	EBL (ml)	Hospital stat (days)	Complications	Bladder biopsy pathology	Postoperative pathology	Follow-up period (mons)
Patient 1	115	45	14	–	Polypoid hyperplasia	Myofibroblastoma	16
Patient 2	125	20	16	Hematuria	Granulation tissue	Adenomatiodmetaplasia	14
Patient 3	120	24	13	–	Inflammatory polyp	Inflammatory granuloma	13
Patient 4	105	15	12	–	–	Urachal cyst	10

## Discussion

In this group, 1 cystoscope biopsy was consistent with the postoperative pathology report, and 2 were inconsistent. Goluboff advocated that preoperative cystoscope biopsies should be performed in order to confirm the diagnosis before treatment. However, due to the deep location of the tumor, it is difficult to obtain ideal lesion specimens for muscular layer and submucosal lesions, which thus results in a low positive rate [1, 2].The pathological bladder tumor types in children are different from those in adults. Most of the tumors in children are non-epithelial in origin,and the bladder surface mucosa is not infiltrated in the early stages. It is difficult to pick up the submucosal tissue using cystoscope biopsy forceps. Despite there being limitations for cystoscopy in children, the size of the lump, its location, and the relationship it has to the ureteral orifice can be intuitively known before surgery, thereby enabling the evaluation of whether a partial resection can be performed and whether the original ureteral orifice can be retained. In addition, rapid pathology can be added during surgery to clarify the pathological type. Ureteral replantation may be necessary during the surgery when the tumor is closely connected to the location of the ureteral orifice detected prior to surgery. However, it also increases the surgical difficulties, and much attention shall be paid to avoid damaging the spermaduct or oviduct.

Rhabdomyosarcoma of the bladder is the main pathological type of pediatric malignant bladder tumor, and it is usually found in the trigone of the bladder, the bladder neck or the prostate urethra. However, most pediatric benign bladder tumors grow outside the trigone of the bladder [3]. In this group, there were 2 cases on the lateralbladder wall, 1 case on the superior wall and 1 case on the inferior wall. Laparoscopic partial cystectomy is suitable for those tumors located at the top or side of the bladder wall but that do not infiltrate the bladder wall. Laparoscopic partial cystectomy is available for only a few patients with localized tumors on the top or lateral wall of the bladder. It is still controversial whether laparoscopic partial cystectomy for the treatment of bladder rhabdomyosarcoma can bring about the risk of intra-abdominal tumor implantation as the surgery is performed through the abdominal cavity and urine is infiltrated into the abdominal cavity after surgery.

The urinary irritation symptoms of the pediatric benign bladder tumor were not obvious, the symptoms appeared late, the tumor infiltrated the muscle layer earlier, and the tumor grew very quickly. The medical history in this group was 7–15 days, with an average time of 10 days, while the tumor size was 2.5–4.5 cm, with an average size of 2.6 cm. In addition, the CT indicated the possibility of a thickening and infiltration of the muscular layer. Due to the size of the tumor and its degree of infiltration, the opportunity of carrying out a transurethral resection was missed. However, laparoscopic partial cystectomy has the advantages of being less traumatic, resulting in less bleeding, being a precise operation, leaving a small scar and having a quick postoperative recovery time. All of which make it suitable for the treatment of pediatric bladder tumors [4–6].Therefore, laparoscopic partial cystectomy was adopted for this group of patients.

The LPC reported in both domestic and foreign literatures is to incise the bladder wall from the top of the bladder, and then incise the bladder in the direction of the tumor slantwise. The operation presents the following problems however: first, when incising the bladder wall, the tumor may also be incised; second, when incising the tumors on the opposite side wall, especially those tumors with a smaller diameter, the bladder incision can come from the middle and becomes large, which then increases the difficulty of suturing in the cavity [7–9]. By performing the 4 cases of laparoscopic partial cystectomy in children, we observed the following techniques and gained this experience:

1. The bladder should be moderately filled, preferably to the pubic symphysis, and the muscle layer of the bladder should be in a certain diastolic state, so as to distinguish the tumor boundary and determine the extent of the surgical resection. Ureteral stents should also be placed bilaterally to protect the ureteral opening.
2. Tumor location: (1) an exogenous tumor’s position can be identified under direct vision. (2) An infiltrative tumor can be located according to the degree of muscle layer thickening, or can be observed by opening the bladder and putting a lens in, whereby the location, size, and the relationship with the bilateral ureteral openings can be directly observed. (3) Using the double lens combination method, whereby a cystoscope is placed through the urethra at a distance of about 0.5 cm from the edge of the tumor. The brightness of the laparoscopic light source is reduced, and the location of the light source of the cystoscope is confirmed, whereupon it should be marked with an electric hook.
3. Bladder suturing is difficult in this operation, and the key points to prevent postoperative urinary fistula are as follows: (1) use a reliable suture technique, such as continuous varus suture. The technical difficulty lies in tightening the knot. 4–0 barbed thread can be used, or it can be knotted every 4 needles, and then embedded with an intermittent suture on the seromuscular layer. (2) The outer wall of the bladder at the site of the tumor should be dissociated in an appropriately large area so that the incisal margin can be easily sutured without tension. (3) Check for leakage by injecting water into the bladder.

Robot-assisted laparoscopic cystectomy in children has been reported in a few literatures [10, 11].Compared with conventional laparoscopy, robot-assisted laparoscopes offer wider and clearer 3D surgical fields for both eyes, allowing to more accurate and flexible control capabilities. In particular, the bladder is located in the pelvic floor in a deeper position, and the procedure performed in such a limited space gives full play to the flexible advantages of robot-assisted surgery; The robot is equipped with special surgical instruments, which can perform wrist-like motions, avoiding subtle tremors, thereby realizing precise and stable sutures. Therefore, the requirements of a skilled surgeon who masters the suture technique are lower than those of conventional laparoscopy, and the suture of bladder reconstruction can be easily accomplished. Compared with conventional laparoscopy, the robot-assisted laparoscopy still has some shortcomings. The introduction and application of the Da Vinci system and related devices are high in cost, bringing about a certain economic burden to medical centers and families; The operating system is not configured with stress feedback, and a surgeon must adjust the strength based on vision and experience; Compared with the 5 mm Trocar of conventional laparoscopy, most of the robots require a larger incision for the 8 mm special Trocar, which leaving in longer scars. In general, as the robot-assisted surgery may further improve the surgical effect, we will try such surgery in future cases.

## Conclusions

The preliminary experience of these 4 cases of laparoscopic partial cystectomy showed that laparoscopic partial cystectomy is suitable for the treatment of pediatric benign bladder tumors and pediatric urachal cysts, and that it is a safe and feasible method. Laparoscopic partial cystectomy has the advantages of being minimally invasive, resulting in less blood loss, having a rapid recover time, only needing a short hospital stay, and causing less postoperative pain. It is a brilliant surgical procedure, which can be used an improved treatment method for pediatric urachal cysts and pediatric benign bladder tumors.

## Data Availability

The data in the study is transparent and open. The data sets used and/or analyzed during the current study are available from the corresponding author on reasonable request.

## Abbreviations

RMS: Rhabdomyosarcoma
CT: Computed tomograp

## References

[1] Lyons TL, Lee T, Winer WK (1998). Lapamseopie removal of a bladder leiomyoma. J Am Asset Gyneeol Laparom.

[2] Nerli RB, Reddy M, Koura AC (2008). Cystoscopy-assisted laparoscopic partial cystectomy. J Endourol.

[3] Diaeonescu S, Burlea M, Miron I (2013). Childhood rhabdomyosarcoma. Anatomo-clinical and therapeutic study on 25 cases. Surgical implications. Rom J Morphol Embryol..

[4] Ahmed H, Howe AS, Dyer LL (2017). Robot-assisted laparoscopic urachal excision in children. Urology.

[5] Kumar S, Shankaregowda SA, Chandna AA (2018). Rare inflammatory myofibroblastic bladder tumor masquerading urachal carcinoma. Urol Ann.

[6] El-Tholoth HS, Al Rasheedi S, Alharbi F (2018). Paraganglioma of urinary bladder managed by laparoscopic partial cystectomy in conjunction with flexible cystoscopy: a case report. J Endourol Case Rep.

[7] Cheah PL, Loo LM, Lee GE, Teoh KH, Mun KS, Nazarina AR (2011). Unusual finding of endocervical-like mucinous epithelium in continuity with uroepithelium in endocervicosis of the urinary bladder. Diagn Pathol.

[8] Olivia Vella JE, Nair N, Ferryman SR, Athavale R, Latthe P, Hirschowitz L (2011). Mullerianosis of the urinary bladder. Intern J Surg Path.

[9] Prager M, Wilson T, Krüger K (2012). Laparoscopic extramucosal partial bladder resection in a patient with symptomatic deep-infiltrating endometriosis of the bladder. J Minim Invasive Gynecol.

[10] Li P, Zhou H, Cao H, Ma L (2020). Robot-assisted laparoscopic radical cystectomy and sigmoid orthotopic neobladder reconstruction for a bladder rhabdomyosarcoma child: case report and literature review. Urology.

[11] Williams CR, Chavda K (2015). En bloc robot-assisted laparoscopic partial cystectomy, urachal resection, and pelvic lymphadenectomy for urachal adenocarcinoma. Rev Urol.

